# Renewing the momentum for leptospirosis research in Africa

**DOI:** 10.1093/trstmh/trv072

**Published:** 2015-09-11

**Authors:** Kathryn J. Allan, Jo E. B. Halliday, Sarah Cleaveland

**Affiliations:** Boyd Orr Centre for Population and Ecosystem Health, Institute of Biodiversity, Animal Health and Comparative Medicine, University of Glasgow, Glasgow G12 8QQ, UK

**Keywords:** Africa, Fever, *Leptospira*, Leptospirosis, Weil Disease, Zoonoses

Leptospirosis is one of the most widespread and pervasive zoonotic bacterial diseases worldwide. Current global estimates indicate that more than 500 000 cases of human disease occur annually and that under-reporting is likely to be substantial especially in endemic regions.^1^ Human leptospirosis, caused by infection with pathogenic *Leptospira* bacteria, can range from a mild, self-limiting or subclinical infection, to a severe, life-threatening disease.^2^ Patients typically present with sudden onset, non-specific febrile illness, which progresses to a severe, life-threatening disease in approximately 10% of cases. Multiple organ systems can be affected and disease manifestations include icterus, renal failure and haemorrhage (Weil's disease), severe pulmonary haemorrhage syndrome (SPHS) or meningitis. The reported case fatality ratio for icteric disease ranges from 5–15% but estimates are higher (>50%) for patients with SPHS.^3^

*Leptospira* infection occurs following direct or indirect contact with the urine of an infected animal. More than 250 identified pathogenic serovars have been identified that can infect a wide variety of mammal species, ranging from domestic animals to rodents and other wildlife.^3^ Leptospirosis is also an important veterinary problem that is often overlooked. Infection in production animals, especially cattle and pigs, can lead to abortions, infertility, and milk and meat production losses.^4^ As a zoonotic disease, the epidemiology of human and animal leptospirosis are intimately linked. However, determining the sources and transmission routes for human infection can be challenging, especially where multiple potential animal hosts co-exist. Environmental persistence of *Leptospira* bacteria further complicates the epidemiology of leptospirosis, particularly in tropical areas where climatic conditions favour prolonged survival.^3^ Disease outbreaks have been associated with heavy rainfall and flooding, poor sanitary conditions in slum environments, and following water-sport events held in contaminated water sources.^5^

In tropical regions of the world, including South-East Asia and South America, leptospirosis is recognised as an important cause of human febrile illness.^2^ In contrast, there is little awareness of leptospirosis in Africa, where climatic and environmental conditions are also predicted to support zoonotic disease transmission. Leptospirosis has not always been neglected in Africa and the disease has a fascinating history on the continent. Following the discovery of leptospirosis in Japan in the early twentieth century, Africa became a hotspot of leptospirosis research as microbiologists worked to differentiate the causal agents of Weil's disease and yellow fever (flavivirus).^6^ In the mid-twentieth century, a group of prolific European researchers, including Van Riel in Belgian Congo,^7^ Mailloux in Morocco^8^ and De Geus in Kenya^9^, generated a wealth of data on human and animal leptospirosis on the continent that has remained unrivalled in either scope or volume. Despite the technological and logistical challenges faced by these researchers, many *Leptospira* serovars of African origin were first isolated and identified during this period.

Unfortunately, current research into leptospirosis in Africa is relatively fragmented with little to no recent data available on human disease in many countries.^10^ Poor public health infrastructure coupled with conflict and economic or political instability have inevitably contributed to gaps in surveillance, particularly from Western and Central Africa. Challenges that limit our ability to detect and diagnose leptospirosis in low-resource settings have further restricted disease surveillance efforts. Little is known about risk factors for endemic infection in either human or animal populations. Laboratory diagnosis of infection can be challenging even in high-income settings, and may not be feasible in under-resourced hospitals and laboratory facilities. Furthermore, acute leptospirosis is often clinically indistinguishable from other causes of febrile illness in tropical areas, such as malaria or dengue fever, and is rarely considered as a differential diagnosis by clinicians. Studies in Tanzania have demonstrated that leptospirosis is an important cause of severe febrile illness in the region but is typically diagnosed and treated as malaria.^11,12^ Given that fever is one of the most common reasons for patients to seek health care in Africa, misdiagnosis of leptospirosis could represent a major public health challenge.

Promoting clinician awareness of leptospirosis is one of the most essential and achievable interventions for tackling leptospirosis in Africa. Leptospirosis is treatable with antimicrobials, including those recommended in international guidelines for the empirical treatment of severe febrile illness in low-resource areas. Therefore, improving clinician awareness of the differential diagnoses for febrile illness, and treatment recommendations for malaria-negative febrile patients, could have a real impact on the care of leptospirosis patients in the future.^13^ Preventive measures in animal populations need to be considered and assessed alongside improved human case recognition and management. *Leptospira* infections are widespread in livestock in Africa,^10^ and livestock vaccinations do exist for some serovars. Effective vaccination use in high-risk animal populations could potentially generate substantial benefits for both human and animal health by reducing *Leptospira* infection in animal source populations, minimising livestock productivity losses and protecting vulnerable farming communities with limited access to health services.^14^

Raising the profile of leptospirosis in Africa with national and international policymakers is also of paramount importance to tackle this neglected but important public health problem. WHO Leptospirosis Burden Epidemiology Reference Group (LERG) and the Global Leptospirosis Environmental Action Network (GLEAN) are working to provide a robust global evidence base to policymakers and researchers. The International Leptospirosis Society, which holds its 9^th^ Biennial meeting in Indonesia (7–10 October 2015; http://ils2015.centrid.org/), plays an important role in disseminating research findings and promoting disease awareness. The International Leptospirosis Society provides invaluable support for leptospirosis researchers around the world with growing interest and enthusiasm for supporting work in Africa.

To understand and quantify the burden and impact of leptospirosis in Africa, we need to rekindle our interest in integrated human and animal leptospirosis research on the continent. As a zoonotic disease, the control of human leptospirosis is unlikely to be effective without also considering control in animal populations. Encouragingly, the ‘One Health’ ideology, which brings together researchers from the fields of medicine, veterinary medicine, sociology and environmental science, is being embraced with enthusiasm in Africa.^15^ With African researchers poised to lead a global trend in zoonotic disease research, exciting opportunities exist to reverse the neglect of leptospirosis in the African continent.
